# Supplementary material for: Cannabidiol Content and In Vitro Biological Activities of Commercial Cannabidiol Oils and Hemp Seed Oils
Source: Medicines (Basel). 2020 Sep 7;7(9):57. doi: 10.3390/medicines7090057 (PMC7555937; doi:10.3390/medicines7090057)
Supplement: Supplementary file 1 [file medicines-07-00057-s001.pdf]

Supplementary Materials: Cannabidiol Content and In Vitro Biological Activities of Commercial Cannabidiol Oils and Hemp Seed Oils

Masashi Kitamura, Yuka Kiba, Ryuichiro Suzuki, Natsumi Tomida, Akemi Uwaya, Fumiyuki Isami and Shixin Deng

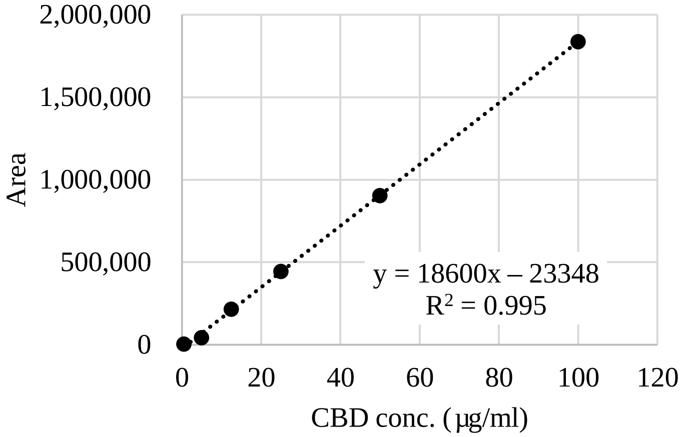

(**a**)

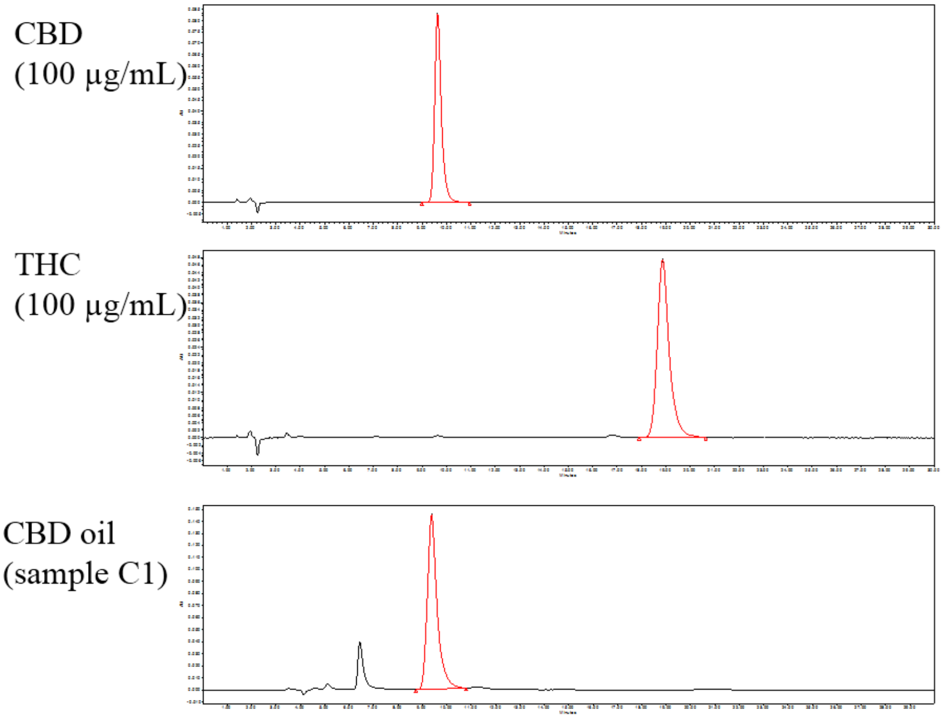

(**b**)

Figure S1. (**a**) Linearity plot of CBD standard. (**b**) HPLC chromatographs of CBD standard, THC standard, and CBD oil sample (C1). CBD and THC reference standards were obtained from Cayman Chemical, Michigan, USA and Sigma-Aldrich, USA, respectively. The CBD concentrations in the CBD and hemp oil samples are shown in Figure 1. THC was not detected in any samples.
